# A 48-channel receive array coil for mesoscopic diffusion-weighted MRI of *ex vivo* human brain on the 3 T connectome scanner

**DOI:** 10.1016/j.neuroimage.2021.118256

**Published:** 2021-06-09

**Authors:** Alina Scholz, Robin Etzel, Markus W. May, Mirsad Mahmutovic, Qiyuan Tian, Gabriel Ramos-Llordén, Chiara Maffei, Berkin Bilgiç, Thomas Witzel, Jason P. Stockmann, Choukri Mekkaoui, Lawrence L. Wald, Susie Yi Huang, Anastasia Yendiki, Boris Keil

**Affiliations:** a Institute of Medical Physics and Radiation Protection (IMPS), TH-Mittelhessen University of Applied Sciences (THM), 14 Wiesenstrasse, Giessen 35390, Germany; b A.A. Martinos Center for Biomedical Imaging, Department of Radiology, Massachusetts General Hospital, Boston, MA, USA; c Harvard Medical School, Boston, MA, USA; d Harvard-MIT Division of Health Sciences and Technology, Cambridge, MA, USA; e Center for Mind, Brain and Behavior (CMBB), Marburg, Germany

**Keywords:** Magnetic resonance imaging, Diffusion-weighted imaging, RF coil, Receive array coil, Brain imaging, Ex vivo brain

## Abstract

In vivo diffusion-weighted magnetic resonance imaging is limited in signal-to-noise-ratio (SNR) and acquisition time, which constrains spatial resolution to the macroscale regime. *Ex vivo* imaging, which allows for arbitrarily long scan times, is critical for exploring human brain structure in the mesoscale regime without loss of SNR. Standard head array coils designed for patients are sub-optimal for imaging *ex vivo* whole brain specimens. The goal of this work was to design and construct a 48-channel *ex vivo* whole brain array coil for high-resolution and high *b*-value diffusion-weighted imaging on a 3T Connectome scanner. The coil was validated with bench measurements and characterized by imaging metrics on an agar brain phantom and an *ex vivo* human brain sample. The two-segment coil former was constructed for a close fit to a whole human brain, with small receive elements distributed over the entire brain. Imaging tests including SNR and G-factor maps were compared to a 64-channel head coil designed for *in vivo* use. There was a 2.9-fold increase in SNR in the peripheral cortex and a 1.3-fold gain in the center when compared to the 64-channel head coil. The 48-channel *ex vivo* whole brain coil also decreases noise amplification in highly parallel imaging, allowing acceleration factors of approximately one unit higher for a given noise amplification level. The acquired diffusion-weighted images in a whole *ex vivo* brain specimen demonstrate the applicability and advantage of the developed coil for high-resolution and high *b*-value diffusion-weighted *ex vivo* brain MRI studies.

## 1. Introduction

Diffusion MRI (dMRI) is a powerful, non-invasive technique for imaging axonal orientations as well as characterizing white and gray matter microstructure (Conturo et al., 1999; Lagana et al., 2010; McNab et al., 2009; Mori and Zhang, 2006; Okano and Mitra, 2015). The basic premise of dMRI in the human brain is that the diffusion of water molecules in white matter is anisotropic, and that its preferential direction is aligned with the orientation of the underlying fibers (Mori and Zhang, 2006). A series of images, each sensitized to diffusion in a different direction, are acquired and used to infer the most likely orientation of water displacement in every voxel (Basser et al., 1994).

There are several requirements that increase the acquisition time needed for whole-brain dMRI. High spatial resolution is desirable for resolving small brain structures. A large number of diffusion directions must be sampled to improve the angular resolution, *i.e.,* the smallest angle between crossing fiber bundles that can be resolved. Finally, advanced dMRI sampling schemes may require images to be acquired with multiple *b*-values. Satisfying all these requirements would lead to acquisition times that are prohibitive for *in vivo* imaging in the absence of any image acceleration. As a result, trade-offs must be made that restrict *in vivo* whole-brain dMRI to the macroscale regime (Okano and Mitra, 2015; Zeng, 2018), with voxel sizes on the order of 1 to 3 mm. Motion artifacts, which are exacerbated by long acquisitions, and distortions near tissue-air interfaces further degrade the effective resolution that is achievable *in vivo*.

Many of these issues can be circumvented in *ex vivo* dMRI, which allows for longer acquisition times, absence of motion and significantly reduced susceptibility artifacts with appropriate sample preparation (Roebroeck et al., 2019). Furthermore, *ex vivo* imaging enables the placement of coil elements closer to the actual brain tissue to maximize sensitivity. Thus, *ex vivo* imaging can achieve substantially higher spatial and angular resolution, permitting the anatomy and microstructure of complex fiber pathways to be imaged at the mesoscale, sub-millimeter regime, well beyond what is feasible *in vivo*. The impressive level of anatomical detail that can be resolved by *ex vivo* dMRI has already been demonstrated on a variety of human tissue samples (Augustinack et al., 2010; Beaujoin et al., 2018; Fritz et al., 2019; Miller et al., 2011; Modo et al., 2016). *Ex vivo* dMRI, in combination with optical imaging, is an excellent tool for validating dMRI acquisition and analysis methods in human brain tissue (Jones et al., 2020; Mollink et al., 2017).

However, various challenges arise when acquiring *ex vivo* dMRI. These primarily include reduced diffusivity and decreased T2, caused mainly by the fixation, tissue dehydration and lower probe temperature (D’Arceuil et al., 2007; Pfefferbaum et al., 2004; Roebroeck et al., 2019). As a result, dMRI data must be acquired with higher *b*-values *ex vivo* to achieve similar diffusion contrast as *in vivo.* Furthermore, when a conventional *in vivo* head coil is used, it is challenging to center the *ex vivo* brain in the coil and to ensure that it remains stable throughout the long scan time.

In addition to the above challenges, the higher spatial resolution of *post mortem* scans comes at the cost of lower signal-to-noise-ratio (SNR). Several strategies for improving SNR in high-resolution *ex vivo* dMRI have been proposed and tested, mainly focusing on higher magnetic field strengths (Pallebage-Gamarallage et al., 2018; Plantinga et al., 2016), small-bore MRI scanners (Augustinack et al., 2010; Calamante et al., 2012) or high-performance gradient systems (McNab et al., 2013). One of the main innovations introduced by the NIH Blueprint Human Connectome Project was the development of human scanners with ultra-high gradients, which allow high *b*-values to be achieved without loss of SNR (Setsompop et al., 2013). Initial results have already shown the advantages of a 300 mT/m gradient system for imaging whole post-mortem human brains at 0.6 mm isotropic resolution (McNab et al., 2013), or smaller, non-human primate brain samples at 0.8 mm isotropic resolution (Eichner et al., 2020). Those results were obtained with an *in vivo* head coil. Dedicated *ex vivo* brain coils are known to increase signal reception sensitivity, and a few studies have shown the benefits of multi-channel brain array coils for *ex vivo* tissue imaging applications (Edlow et al., 2019; Roebroeck et al., 2015; Sengupta et al., 2018).

The aim of this study was to push the limits of spatial and angular resolution in *ex vivo* dMRI by designing, constructing, and validating a 48-channel (48ch) receive array coil for *ex vivo* whole human brain examinations. The array coil was developed for high spatial resolution and high *b*-value dMRI acquisitions with long scan times (a few hours to a few days) on the 3 T Connectome scanner (McNab et al., 2013; Setsompop et al., 2013). This work presents high-sensitivity *ex vivo* diffusion MRI results obtained in a whole human brain specimen at mesoscale resolution (0.73 mm isotropic) using the 48ch receive coil on the 3 T Connectome scanner and expands on preliminary results that were published in conference proceedings (Scholz et al., 2019).

## 2. Material and methods

### 2.1. Coil design and construction

**Fig. 1.**
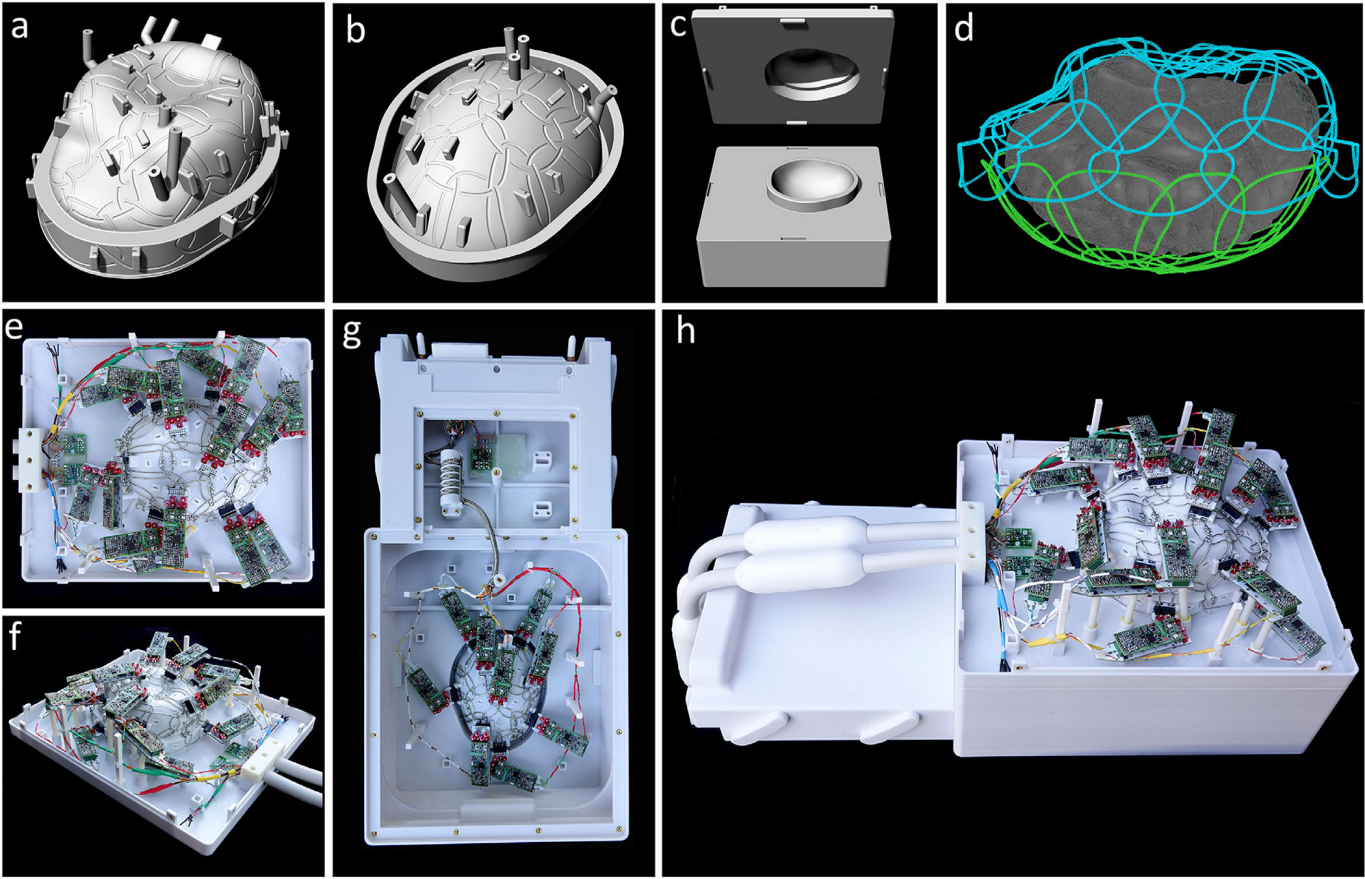
48ch *ex vivo* brain coil **(a-c)** Computer aided design model of the coilformer with graved loops and standoffs for preamplifier boards. **(a)** Top coilformer part. **(b)** Bottom coilformer part. **(c)** Inner side of both coilformer parts with overlapping frames to allow geometrical decoupling of the loops from top and bottom part. **(d)** Placement of the 30 top loops (blue) and the 18 bottom loops (green) around the brain (gray). **(e-h)** Completely constructed coil consisting of the top part (e and f) and bottom part (g).

To closely cover a whole human brain, we designed an anatomically-shaped *ex vivo* brain coil former (Fig. 1a and b) based on a nonlinear brain atlas of the International Consortium for Brain Mapping (ICBM). The coil housing was modeled with 3D computer aided design (CAD) software (Rhino3D, Robert McNeel & Associates, Seattle, WA, USA, version 6). It was designed to completely surround the brain with minimal space between the coil elements and imaging volume. The coil former was split into an upper and lower part, such that a whole brain can be placed inside the coil container. Both coil segments close with an overlapping rim structure (Fig. 1c). The coil container can accommodate whole brains with dimensions of 140 mm in the left-to-right direction, an anterior-to-posterior diameter of 182 mm, and a superior-inferior distance of 110 mm. The completed array coil is shown in Fig. 1e–h.

In the bottom coil segment, we incorporated the mechanics for a plugging slide mechanism (Fig. 1g and h), which directly plugs it into the scanner’s patient bed. The top coil segment is connected to the scanner using two standard multi-channel coil plugs. The *ex vivo* coil container was designed to allow the brain to be placed at the isocenter of the scanner.

The optimum channel count for the constructed *ex vivo* brain array was determined by (1) the given area of the coil former’s surface, and (2) a suitable coil element’s *Q*-ratio for maintaining sample noise domination. These constraints resulted in a loop count of 48 and a loop diameter of 54 mm, which comprises an inductance of about 203 nH. The positions of the 48 coil elements on the outer surface of the coil former were derived from a hexagonal/pentagonal tiling pattern (Wiggins et al., 2006), with 30 and 18 coil elements located on the top and bottom segments, respectively (Fig. 1d). The position and outline of all loop elements, which are decoupled geometrically from neighboring loops by critical overlap (Roemer et al., 1990), were incorporated in the CAD model. The majority of the loops was circularly formed, whereas some loops were arbitrarily shaped to fit over the rim structure. The critical overlap was determined empirically in previously tested bench measurements and is about 0.27 times the loop diameter. Standoffs for circuit boards and cable routing were implemented to provide stable mounting positions. The coil former including its cover were then 3D-printed in polycarbonate (PC) using a 3D printer (Fortus 350, Stratasys, Eden Prairie, USA).

### 2.2. Coil circuit

The loop elements were constructed out of 1.3 mm thick tin-coated copper wire. Compared to flat circuit board copper traces, the wire loops reduce eddy current losses in a high-density array coil architecture (Kumar et al., 2009). Implemented small bridges in the conductor enable one loop to cross over another without touching (Keil and Wald, 2013).

**Fig. 2.**
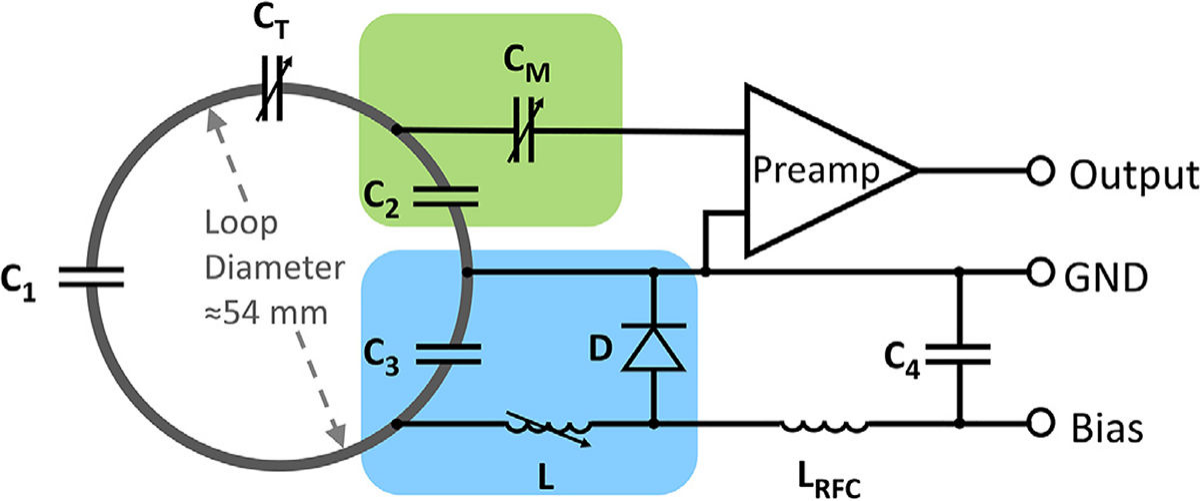
Circuit schematic for one coil element. Each loop consists of three fixed capacitors (*C*_1_–*C*_3_) and one variable capacitor (*C*_*T*_). *C*_*T*_ fine-tunes the resonant frequency of the coil to Larmor frequency corresponding at 3T. *C*_2_ and *C*_3_ create a capacitive voltage divider. *C*_3_ is part of the active detuning circuit (blue) together with the variable inductor *L* and the PIN diode *D*. *C*_2_ and *C*_*M*_ (green) provide both impedance matching of the loop and impedance transformation to establish preamplifier decoupling. Typical values for the components are: *C*_1_ = 33 pF, *C*_2_ = 56 pF, *C*_3_ = 56 pF, *C*_4_ = 2.2 nF, *C*_*T*_ ≈ 18 pF, *C_M_* ≈ 18 pF, *L* ≈ 24.5 nH, *L*_*RFC*_ = 2.7 μH.

Each coil circuit (Fig. 2) consists of a loop with three symmetrically placed ceramic capacitors (Series 11, Voltronics, Danville, NJ), one variable plastic capacitor (GFX2700NM; Sprague Goodman, Westbury, New York, USA), a matching network to the preamplifier (Siemens AG, Healthineers, Erlangen, Germany), and an actively controllable detuning resonant circuit. A typically redundant passive detuning safety mechanism for *in vivo* examinations was omitted for this *ex vivo* coil.

The variable capacitor *C*_*T*_ (3–33 pF) was used to fine-tune the loop resonance to the Larmor frequency at 3T (123.25 MHz). *C*_2_ and *C*_3_ create a capacitive voltage divider. The variable capacitor *C*_*M*_ (3–33 pF, GFX2700NM; Sprague Goodman, Westbury, New York, USA) provides impedance matching of the loop output to a 50 Ω noise matched condition needed by the preamplifier to operate at the lowest noise figure at 123.25 MHz (Reykowski et al., 1995). To ensure accurate detuning of the loop elements, an active detuning circuit was implemented. It consists of one of the voltage dividing capacitors *C*_3_ and a variable inductor *L* (Coilcraft Inc., 25–32 nH, 165-02A06L, Cary, IL, USA) in series to a PIN diode *D* (MA4P4002B-402; Macom, Lowell, MA, USA) (Edelstein et al., 1986). During transmit, a DC current is applied to forward bias the PIN diode. This in turn activates the detuning resonant circuit at the Larmor frequency and generates a high impedance in the loop to suppress current flow. The RF-choke *L*_*RFC*_ (Coilcraft Inc, 2.7 μH, 1812CS-333XJLC Cary, IL, USA) and *C*_4_ block the RF signal to prevent passing into bias source.

While nearest neighbors use geometrical decoupling, next-nearest neighbors and further coil elements are decoupled by the impedance transformation of the input of the preamplifiers (Roemer et al., 1990). The capacitors *C*_2_ and *C*_*M*_ and the preamplifier’s input impedance form a resonant circuit, which enables a voltage-source measurement setup, where RF current flow is minimized. As a consequence, inductive coupling across elements is highly reduced and all coil elements receive independently, while maintaining a 50 Ω output impedance.

Both the matching and detuning network of the coil element are placed on the preamplifier’s daughter board, rather than soldering these components directly to the coil former. Therefore, the daughter board is a part of the coil element. The printed circuit board (PCB) daughter board is connected to the loop with an intermittent pin connector. This setup allows a fast construction process of dense array coils.

According to the RF scanner architecture, pre-amplified signals from two loops elements are multiplexed onto one output coaxial cable. The bundled output cables are passed through cable traps to prevent RF common mode currents on the shield of the coaxial cable (Peterson et al., 2003). The cable traps comprise a wounded coaxial cable bundle, which form an inductance (≈109 nH), and a parallel ceramic high power capacitor (15.2 pF, Series 25, Voltronics, Danville, NJ), which resonates at Larmor frequency. Two traps are incorporated into the cables of the upper array coil segment and one cable trap is located directly in the bottom coil housing part.

### 2.3. Coil bench measurements

For bench measurements during the construction process, a custom-made coil plug simulator was used. It provides voltage for the preamplifiers (3 V) and the opportunity to apply a DC current (100 mA) to bias manually each PIN diode forward, which allows for active detuning of single coil elements. To gather information about bench level metrics, e.g. transmission and reflection measurements, a vector network analyzer (VNA) (ENA series, Agilent Technologies, Santa Clara, CA) and custom-built RF tools such as single / double probes and sniffer probes were used. These measurements included tuning to Larmor frequency, active detuning, preamplifier decoupling and geometrical nearest neighbor decoupling of each coil element.

The loops were tuned under a *S*_21_ control with a 50 Ω dummy load plugged into the preamplifier socket, while all other elements of the array were detuned. Active detuning was performed by using *S*_21_ measurement with the double-probe for each loop, while all other coil elements were detuned and the relevant loop under test was switched between the tuned and detuned state. The difference of both states at the Larmor frequency indicates the magnitude of active detuning. A similar *S*_21_ double-probe measurement was carried out to determine the effectiveness of the implemented preamplifier decoupling, first by plugging the preamplifier into the socket on the PCB and second by terminating the socket with a load impedance of 50 Ω. Again, all but the loop element to be tested were detuned.

Coupling of nearest neighbor elements was measured with direct *S*_21_ VNA measurement by using coaxial cables, which were directly plugged into the preamplifier sockets. During this measurement, all other coil elements were detuned. This measurement configuration was also used to verify 50 Ω coil impedance matching using *S*_11_ and *S*_22_ measurements (Keil and Wald, 2013; Reykowski et al., 1995).

Furthermore, unloaded-to-loaded coil quality factor ratio (*Q*_*U*_/*Q*_*L*_) of one representative coil element was measured within the populated but detuned array assembly, using the *S*_21_ double-probe method (Hoult, 1978). As a load, a fixed tissue brain sample in periodate-lysine-paraformaldehyde (PLP) solution was used.

### 2.4. MRI data acquisition and analysis

Imaging metrics were acquired on a clinical 3T MRI scanner (MAG-NETOM, Skyra, Tim 4G, Dual Density Signal Transfer, Siemens AG, Healthineers, Erlangen, Germany), equipped with a customized gradient coil (AS302 CONNECTOM 1.0 gradient) ^1^ with a maximum gradient strength of 300 mT/m and a maximum slew rate of 200 T/m/s.

For evaluating the developed *ex vivo* whole brain array coil, we constructed a human-brain-shaped phantom using a 3D printer (Objet30 Pro, Stratasys, Eden Prairie, USA). The phantom was filled with agarose and dielectrically tuned to match the RF coil’s loading condition with the PLP-packed *ex vivo* brain. The corresponding quantities were: 830ml distilled H_2_O, 29 g NaCl, 12.5 g of agar powder (Sigma-Aldrich Corp., St. Louis, MO) and 936 g sugar. The matched loading condition was validated via an *S*_11_ measurement on the VNA’s smith chart at Larmor frequency. The dielectric values of the phantom were measured to be *σ* = 0.49 S/m and *ε*_*r*_ = 66.3 with a VNA equipped with a dielectric probe kit (85070E kit, Agilent Technologies, Santa Clara, CA).

#### 2.4.1. Array coil characterization

For determining SNR and G-factor, the phantom was scanned with a proton density (PD)-weighted FLASH sequence (repetition time (TR)=200 ms, echo time (TE)=4.8 ms, flip angle (F) =15°, matrix (M): 192×192 (SNR) and 64×64 (G-factor and SNR in parallel imaging), field of view (FOV): 256×256 mm^2^, slice thickness: 8 mm, bandwidth (BW): 200 Hz/pixel). Information about noise correlation was obtained with the same sequence but without RF excitation. The coil sensitivities for the G-factor calculations were derived from a pre-scan before the actual MRI data acquisition. This scan provided a low-resolution full FOV image of the phantom for each coil element, which was used to estimate the sensitivity profiles of the individual receiver coil.

Pixel-wise SNR maps were calculated using the noise-covariance-weighted, root sum-of-squares image reconstruction method from Kellman and McVeigh (2005). To evaluate the array coil’s encoding capability for parallel imaging, SENSE G-factor maps were computed using the acquired noise correlation matrix and complex sensitivities of the coil elements (Pruessmann et al., 1999). The FOV of the G-maps was tightly enclosed to the phantom, in order to enhance the aliasing pattern inside the imaging object.

A valuable metric is the remaining image SNR after the parallel imaging acceleration has been performed. We calculated the remaining SNR by dividing the SNR globally by the square root of the reduction factor *R* and further locally with the noise amplification given by the G-factor.

For further characterization of the coil performance, we examined the encoding power for simultaneous multislice (SMS) acquisitions with blipped-controlled aliasing in parallel imaging (Feinberg et al., 2010; Larkman et al., 2001; Setsompop et al., 2012). To assess the encoding capability of combined SMS and in-plane acceleration, a reduction factor of *R* = 2 and a slice acceleration factor from *MB* = 4 up to *MB* = 8 with a 1/3 FOV shift were evaluated. Noise correlation and SNR and G-factor maps of the 48ch *ex vivo* brain coil were compared to a customized 64-channel (64ch) whole head receive array coil (Keil et al., 2013) with identical acquisition parameters.

In addition, time course stability of each coil element was measured with a single-shot, gradient-echo, echo planar imaging (EPI) sequence (time points: 500, TR = 1000 ms, TE = 30 ms, F = 90°, M: 64 × 64, FOV: 200 × 200 mm^2^, slices: 16 slices of 15 mm, BW: 2298 Hz/pixel) with the brain phantom. This scan was repeated 16 times without pause, resulting in a 2 h stability scan protocol. The average intensity of a 15-pixel square region of interest (ROI) in the phantom center was detrended with linear and quadratic temporal trends and plotted. The stability was calculated as the variation of signal intensity from peak-to-peak as a percentage from the average signal intensity (Weisskoff, 1996).

#### 2.4.2. Ex vivo *brain dMRI*

High-resolution (0.73 mm isotropic) diffusion imaging was performed on a whole *ex vivo* human brain packed with paraformaldehyde-lysine-periodate (PLP) in a tight-fitting, sealed plastic bag. The brain had been excised from a male who had died of non-neurological causes, and had been placed in fixative (10% formaldehyde) for 90 days before being transferred to PLP solution for long-term storage. Diffusion-weighted images were acquired using the same imaging protocol on the 48ch whole brain *ex vivo* coil and 64ch *in vivo* head coil to enable comparisons between the two coils. We used a 3D diffusion-weighted spin-echo segmented EPI sequence (imaging parameters: TR = 500 ms, TE = 65 ms, echo spacing: 1.22 ms, M: 160 × 268 × 208, FOV: 118 × 196 × 152 mm^3^, BW: 1244 Hz/pixel, 16 shots, EPI factor = 10, no partial Fourier). A multi-shell sampling scheme was used that included 18 non-collinear diffusion encoding directions with *b* = 4000 s/mm^2^ (gradient strength of 91 mT/m, *δ* = 16.1 ms, Δ = 27.6 ms), and 36 non-collinear diffusion encoding directions with *b* = 10000 s/mm^2^ (gradient strength of 133 mT/m, *δ* = 16.1 ms, Δ = 27.6 ms). A total of 9 *b* = 0 volumes were acquired, interleaved every 6 diffusion-weighted volumes. The total acquisition time for the *b* = 4000 s/mm^2^ scan was 10.3 h. The total acquisition time for the *b* = 10000 s/mm^2^ scan was 20.6 h. The phase-encoding direction was anterior-posterior when considering the conventional sagittal plane. Since the brain in the constructed coil was rotated compared to the usual orientation of a patient, the anatomical axis of the phase-encoding direction was inferior-superior.

The image acquisition parameters were chosen so as to maximize SNR while avoiding prohibitively long scan times. The use of a 3D read-out with excitation of the entire imaging volume per TR enabled the use of a relatively short TR of 500 ms while preserving SNR efficiency (Miller et al., 0000). Due to the reduction of *T*_1_ and *T*_2_ values in fixed human brain tissue (340 ms and 45 ms, respectively (McNab et al., 2009)) compared to *in vivo* values, a relatively short TE of 65 ms was chosen to preserve SNR. To achieve the TE and *b*-values used here, the maximum gradient strength was calculated to be on the order of 90–130 mT/m based on the sequence design, which seeks to maximize gradient strength while keeping the diffusion time Δ as short as possible.

Diffusion-weighted volumes were corrected for eddy current distortions with the *eddy* tool from FSL (Andersson and Sotiropoulos, 2016). The multi-shot acquisition mitigated EPI distortions and thus no further distortion correction was performed prior to image analysis. We performed a diffusion tensor imaging (DTI) analysis (Basser, 1995) on the images from the lower shell, and a diffusion kurtosis imaging (DKI) analysis (Jensen et al., 2005) on the images from both shells. Finally, the full data set was used to fit a fiber orientation distribution (FOD) at each voxel with multi-shell, multi-tissue, constrained, spherical deconvolution (MSMT-CSD) (Dhollander et al., Montreal, 2019). Probabilistic tractography was then performed on these FODs (Tournier et al., Stockholm, 2010), with a minimum FOD amplitude of 0.2, a maximum bending angle of 45 degrees, and a minimum streamline length of 20. We seeded at every voxel in a white-matter mask, which we extracted by thresholding the average of the diffusion-weighted volumes to remove the PLP background.

## 3. Results

### 3.1. Coil bench measurements

The *Q*_*U*_*/Q*_*L*_-ratio of a 54 mm loop element was measured to be 233/46=5.1 with six surrounding but non-resonant neighboring loops. Thus, the array’s loop elements operate in the sample noise dominated regime. The geometrical decoupling of nearest neighbors was *S*_*21*_ measured with an average value of −16 dB and ranged from −14 dB to −18 dB. Non-adjacent and thus non-overlapping coil elements, which are primarily decoupled via preamplifier decoupling, obtained an average decoupling value of −18 dB with a range from −17 dB to −19 dB. The isolation between tuned and detuned states caused by the active detuning circuit reached an average value of 42 dB.

### 3.2. Image performance

Time course stability tests show a peak-to-peak variation of 0.36% over 8.000 time-points EPI sequence measured in a ROI comprising 15×15 pixels.

#### 3.2.1. Signal-to-noise-ratio in unaccelerated images

**Fig. 3.**
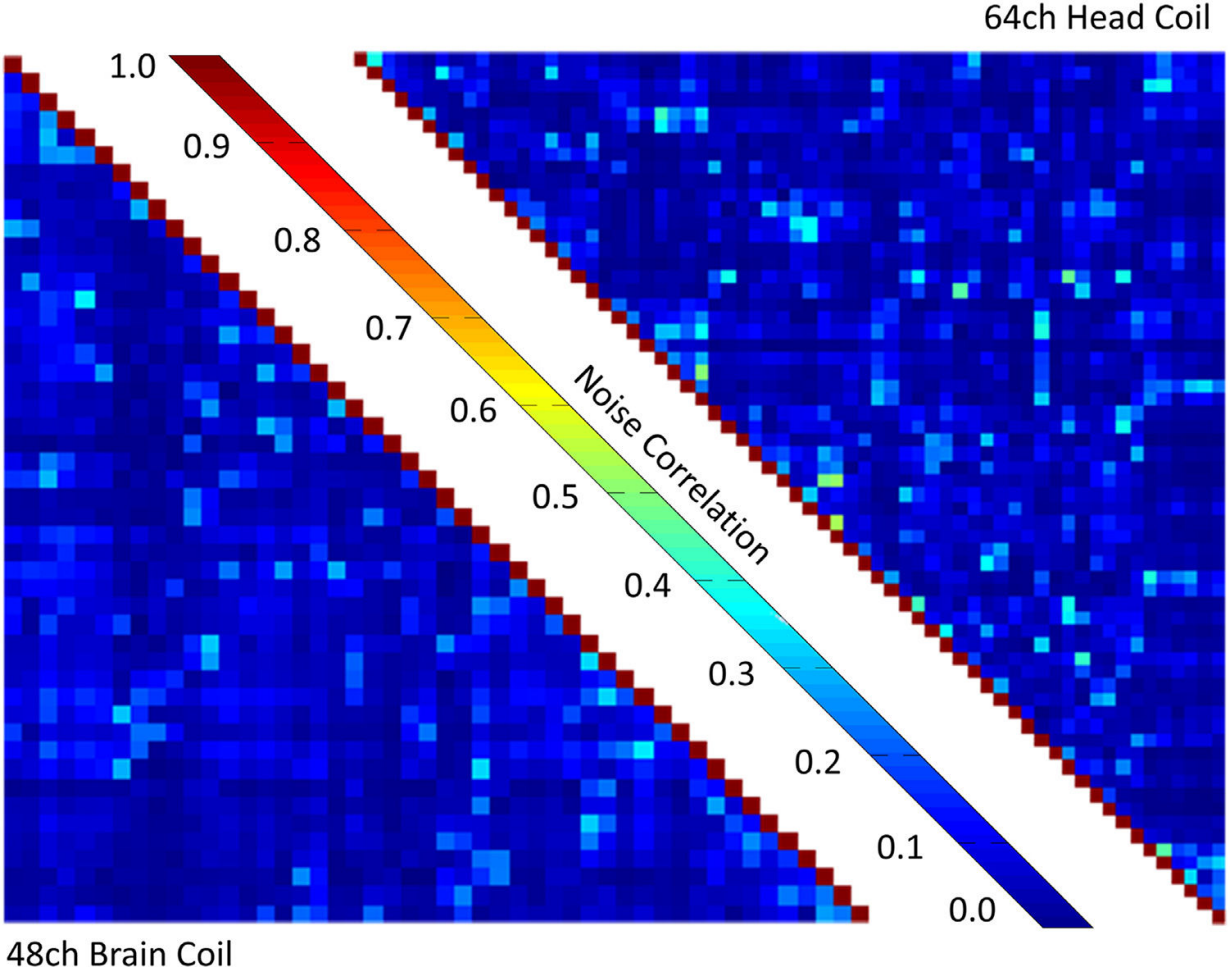
Noise correlation matrix of the 48ch *ex vivo* brain coil and the 64ch *in vivo* head coil with the scale normalized to 1.

Fig. 3 shows the noise correlation matrix of the 48ch *ex vivo* coil and that of the 64ch *in vivo* coil. The *ex vivo* array has a range of noise correlations from 0.02% to 35.8% with an average value of 7.5%, while the *in vivo* array has noise correlations from 0.12% to 53.8% with an average value of 7.1% for the off-diagonal elements.

**Fig. 4.**
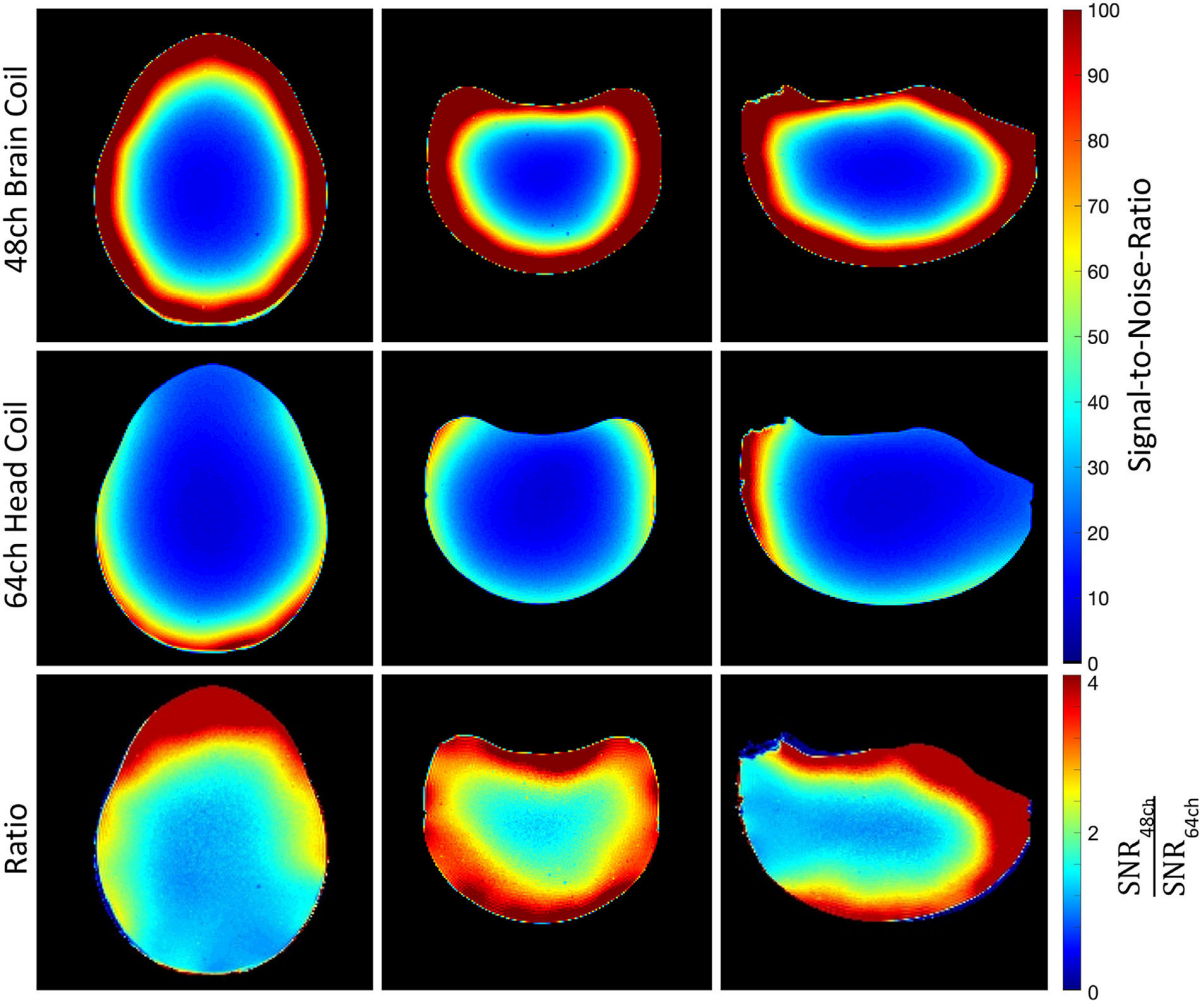
Comparison of the SNR, normalized to 100, of a transverse (left), coronal (middle) and sagittal (right) slice of the brain phantom with the 48ch *ex vivo* brain coil (top row), the 64ch head coil (middle row) and ratio maps between the two coils (bottom row). The 48ch *ex vivo* brain coil shows a 1.3-fold SNR gain in the center and a 2.9-fold SNR improvement in the peripheral regions when compared to the 64ch head coil.

Fig. 4 compares the SNR maps from the newly developed 48ch *ex vivo* brain coil to that of the existing, custom 64ch whole head coil, in different planes of the agar phantom. For both coils, the measured SNR is highest in the outer periphery and decreases towards the center. The SNR gain of the newly constructed 48ch array coil reaches over the whole brain volume. It outperforms the larger 64ch *in vivo* head coil by a factor of 2.5, when the average SNR over the whole brain phantom is considered. The highest gain is found in the periphery of the phantom, especially in the regions where the 48ch brain coil has a substantially closer proximity to the sample. In the periphery and in the center of the phantom, a 2.9-fold and 1.3-fold SNR gain was measured, respectively.

**Fig. 5.**
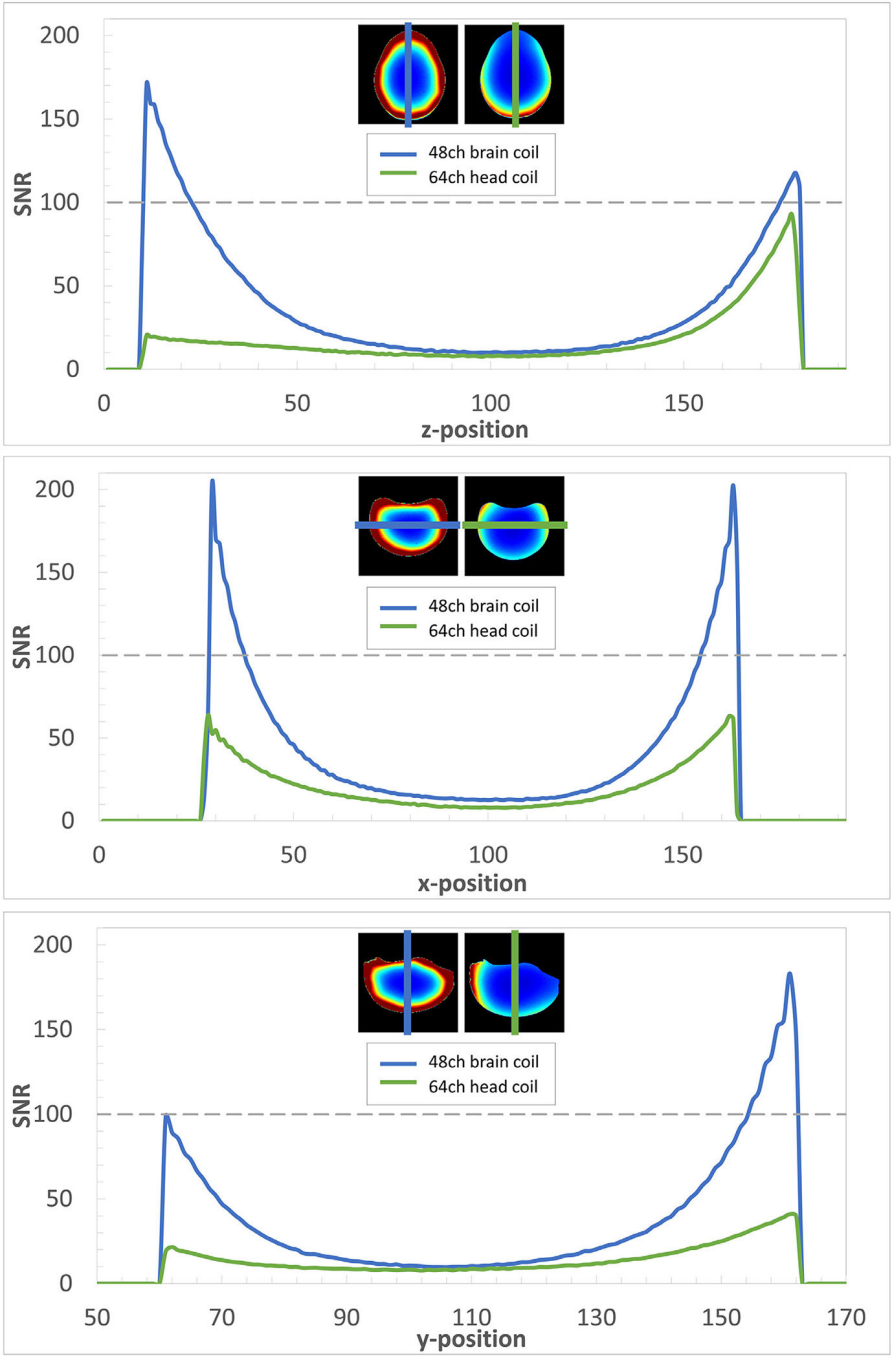
SNR profiles of the 48ch *ex vivo* brain coil (blue) and the 64ch *in vivo* head coil (green) through the center of a transverse (top), a coronal (middle), and a saggital (bottom) slice. The dedicated 48ch *ex vivo* coil shows substantial SNR gains at the periphery in the brain phantom. Due to the close-fitting coil array with omnidirectional signal reception, even SNR improvements at the center of the brain phantom of ≈30% are feasible.

Examples of SNR profiles can be found in Fig. 5. In all three slices, the SNR of the 48ch *ex vivo* brain coil exceeds that of the 64ch *in vivo* head coil over the entire profile.

#### 3.2.2. Signal-to-noise-ratio and G-factor in parallel imaging

**Fig. 6.**
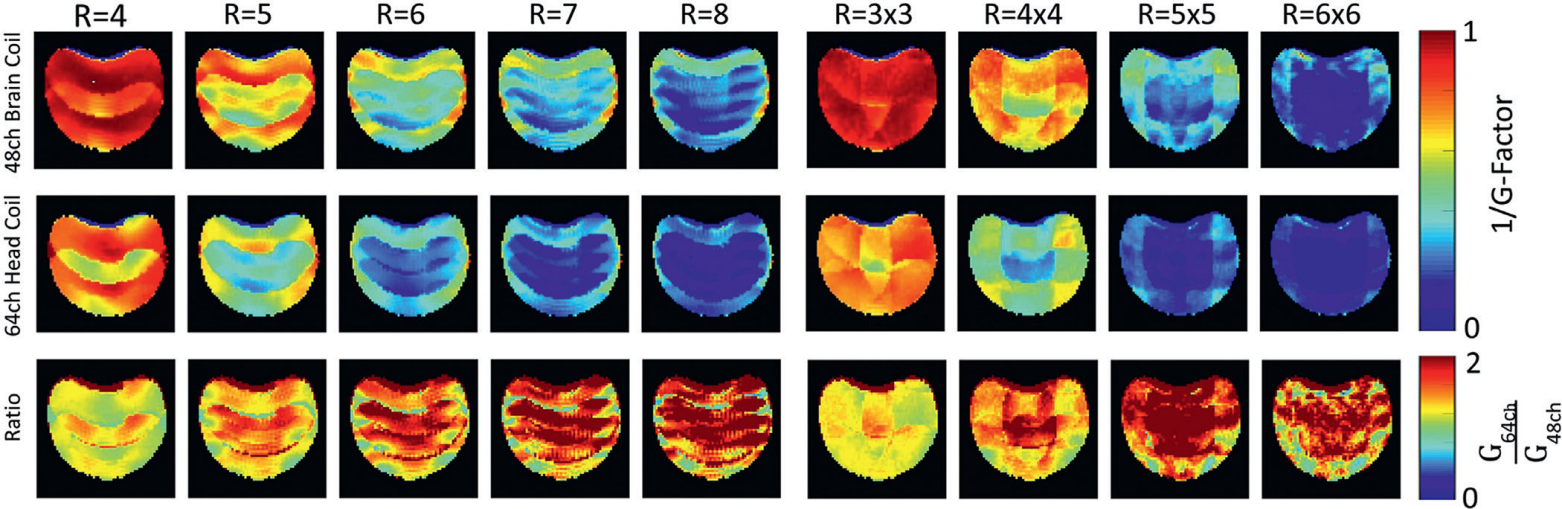
Comparison of inverse phantom G-factor maps between the 48ch *ex vivo* brain coil (top row) and the 64ch head coil (middle row) for different acceleration factors (*R*) obtained from a representative coronal slice. The bottom row shows the ratio maps of the inverse G-factors obtained from both coils. The G-Factors from the 48ch *ex vivo* brain coil show overall lower noise amplification, when compared to the 64ch head coil.

Fig. 6 shows the SENSE inverse G-factor maps in a representative coronal plane of the brain phantom for both one-dimensional and two-dimensional acceleration obtained from the 48ch *ex vivo* brain coil and the 64ch *in vivo* head coil. The newly constructed 48ch coil provides significant improvement compared to the 64ch head coil for both in-plane acceleration types. Both coils show minimal noise amplifications for acceleration factors of *R* = 2, *R* = 3 and *R* = 2×2. However, for higher accelerations (*R*>3) the 48ch *ex vivo* coil provides favorable encoding capabilities when compared to the 64ch *in vivo* head coil. At *R* = 4, the 48ch coil shows on average a 16% lower G-factor than the 64ch head coil. When comparing the peak G-factors between both, the 48ch coil shows a 21% improvement. The enhanced encoding power of the 48ch coil becomes even more apparent when very high acceleration factors are compared. The improved average and peak G-factor for *R* = 7 was measured to be 35% and 41% lower. At *R* = 5×5 the noise amplifications could be reduced on average by 43%, while the peak G-factor decreased by 53%.

**Fig. 7.**
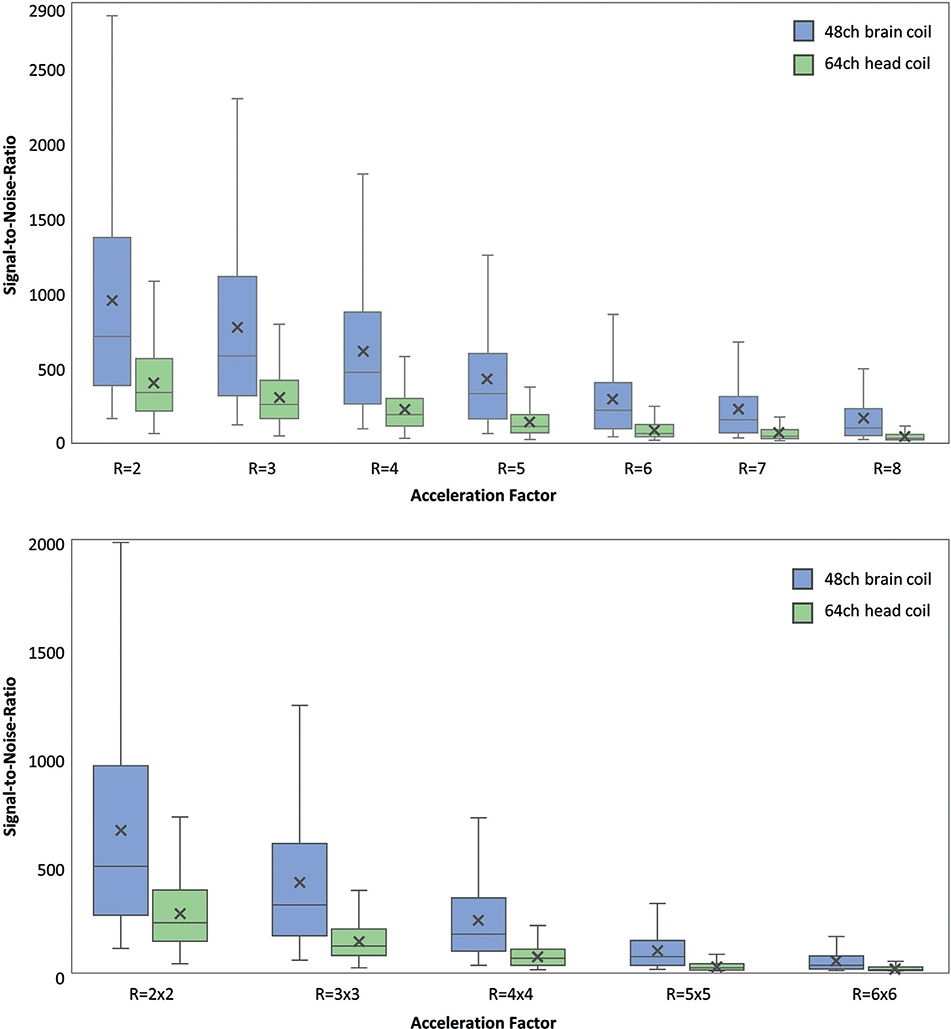
Parallel imaging accelerated SNR as a function of acceleration factor (*R*) from the 48ch brain coil and the 64ch head coil for one-dimensional (top) and two-dimensional (bottom) accelerations. The box plots represent median (horizontal line), average (cross mark) lower/upper quartiles and minimum-maximum range (whiskers) without outliners. The constructed 48ch coil shows higher accelerated SNR in the entire range of acceleration factors.

A more meaningful figure of merit is the SNR obtained from the accelerated image, where both the under-sampled k-space trajectory and the local noise amplification were taken into account. Fig. 7 illustrates the accelerated SNR for both coils using box plots. Since the constructed 48ch coil provides both a higher baseline SNR and lower G-factors, it highly outperforms the 64ch head coil across all acceleration scenarios. The average SNR from the 64ch coil only reaches the lower 25th percentile of the 48ch *ex vivo* coil. Further, it should be noted that the relative gain in average SNR increases with higher acceleration factors (*e.g.,* factor 2.4 for *R* = 2 and 3.9 for *R* = 7) for both one-dimensional and two-dimensional acceleration. For a direct comparison between 1D and 2D accelerations, when the reduction factor *R* was matched (*R*_1D_=4 and *R*_2D_=2×2=4), the average SNR of the 48ch ex-vivo coil was measured to be 600 and 657, respectively. For the 64ch head coil, these numbers were 210 and 268, respectively. Thus, both coils showed overall less noise amplification in the 2D-acceleration scheme.

#### 3.2.3. Signal-to-noise-ratio and G-factor in simultaneous multislice imaging imaging

**Fig. 8.**
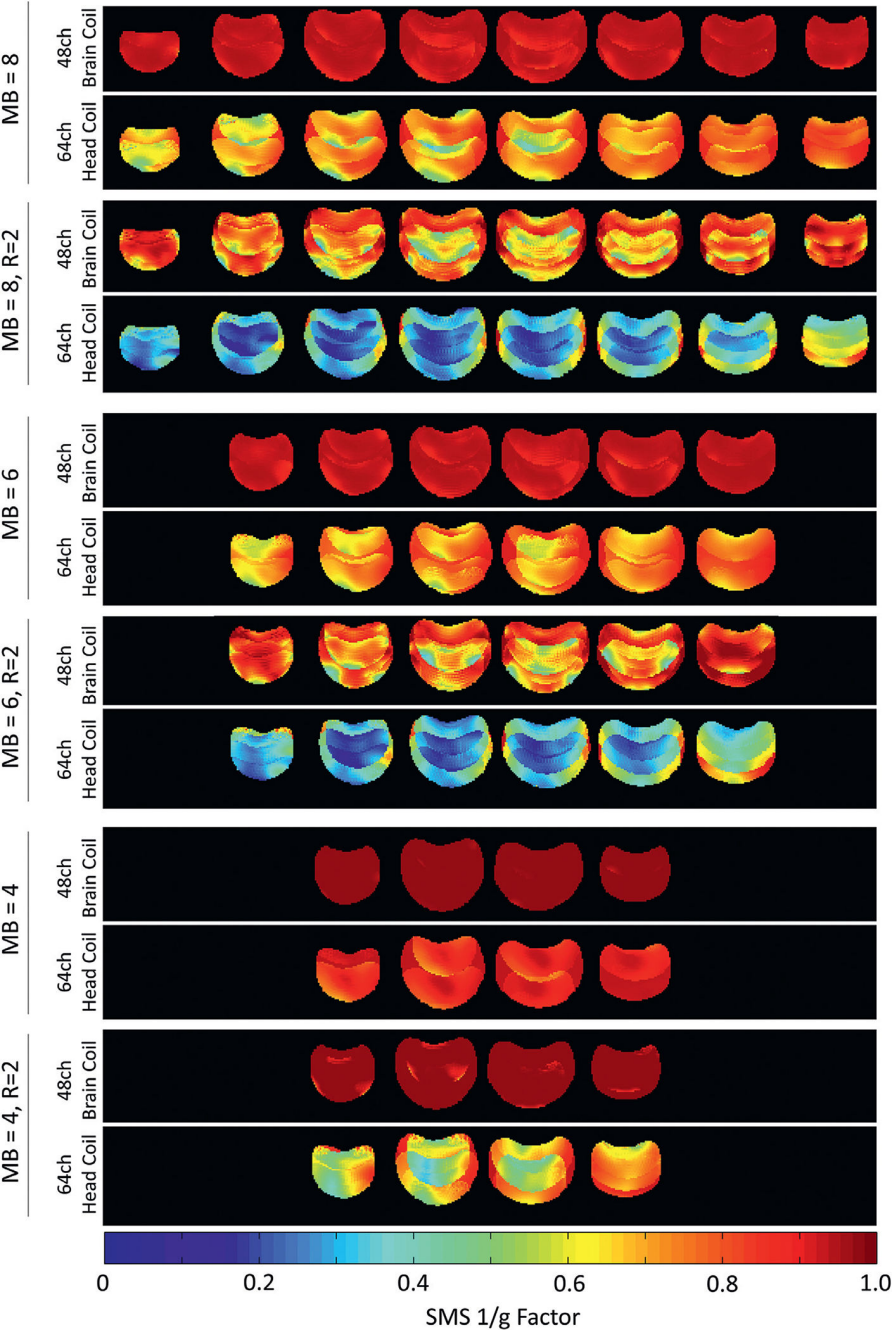
Comparison of inverse G-factor maps of the brain phantom for accelerated imaging with SMS technique. The 48ch brain coil shows overall considerable lower noise amplification in comparison to the 64ch head coil.

Fig. 8 compares the inverse G-factor maps for the SMS image reconstruction technique from a coronal slice of the brain phantom. Compared to the 64ch head coil, the constructed 48ch coil indicates overall substantially lower noise amplification for the SMS examination, as well as for combined SMS and in-plane acceleration. At a multi-band factor of *MB* = 4, the 48ch coil generates negligible noise amplifications (*g*_mean_=1.0002 and *g*_max_=1.0569), while the 64ch head coil shows substantial noise gains of *g*_mean_=1.1218 and *g*_max_=1.6345. Furthermore, the dedicated 48ch *ex vivo* brain coil achieves similar to slightly better encoding capabilities at *MB* = 8 as the 64ch head coil at *MB* = 4 (*g*_mean48ch_=1.0047 *vs. g*_mean64ch_ = 1.1317 and *g*_max48ch_=1.2913 *vs. g*_max64ch_=1.2913). Therefore, the 48ch coil allows the application of a slice acceleration factor of *MB* = 8 with negligible noise gain.

To assess the accelerated SNR during SMS acquisitions, Fourier averaging needs to be taken into account: In the case of the *MB* = 8 acceleration, eight times more ^1^H spins are simultaneously excited compared with a single-slice acquisition. Thus, for a multiband factor *MB*, the SNR efficiency can be improved up to a factor of √*MB*, if the imaging parameters between the non-accelerated and the SMS-accelerated case remain identical. Under these constant circumstances, the Fourier averaging translates to an SNR increase by a factor of up to $\sqrt{8}/g_{\max-48}=2.2$, when compared to a commonly used consecutive single-slice acquisition schemes. The *MB* = 8 achievable SNR obtained from the 64ch is only increased by a factor of up to $\sqrt{8}/g_{\max-64}=2.2$. In direct comparison, when the baseline SNR, Fourier averaging, and G-factors are taken into account, the 48ch coil achieves up to a 4.5-fold SNR improvement at *MB* = 8 compared to the 64ch head coil.

#### 3.2.4. *Diffusion imaging in* ex vivo *brain*

**Fig. 9.**
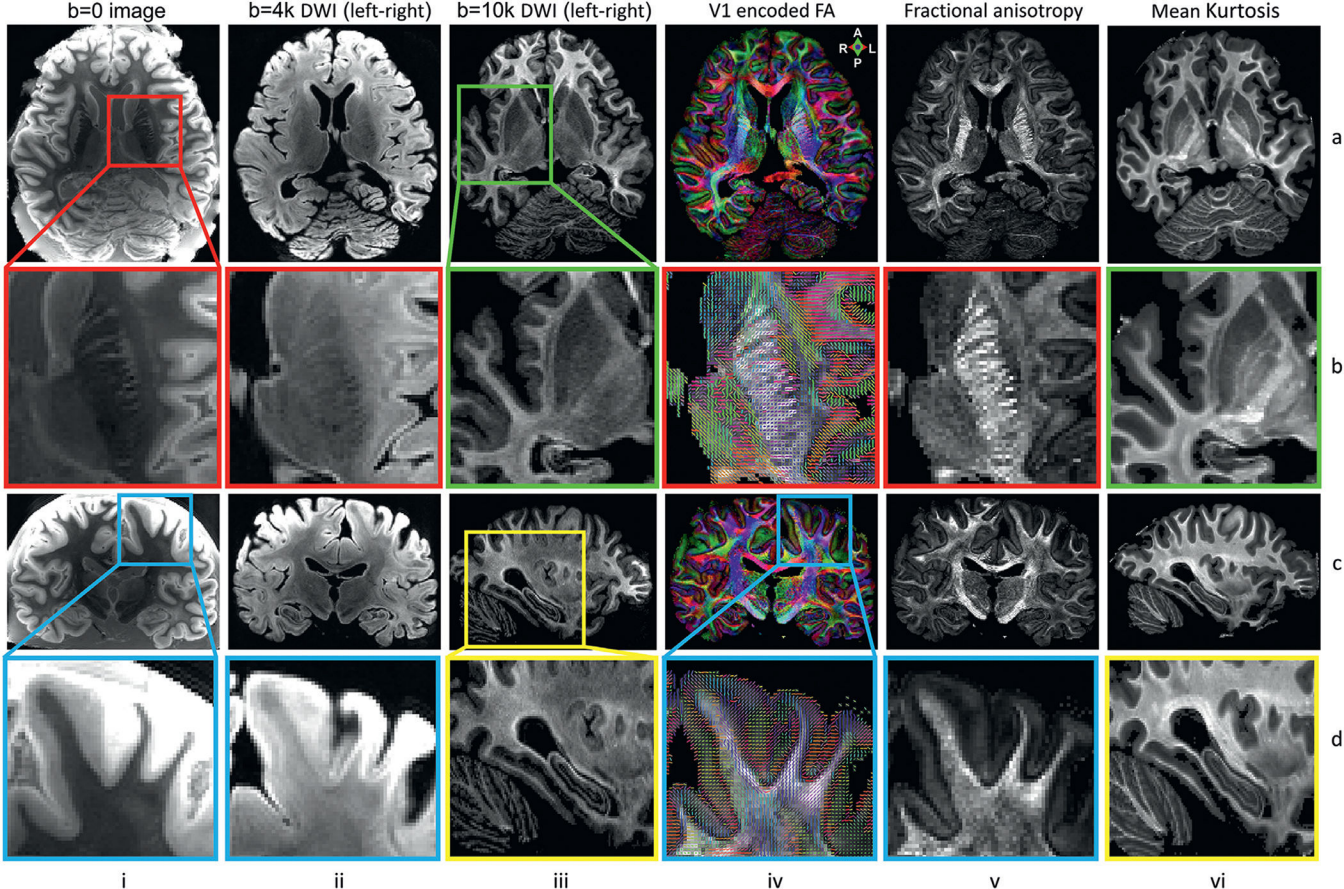
High-resolution DTI and DKI results at 0.73 mm isotropic resolution with *b* = 0 images (column i), diffusion-weighted images (DWI) acquired at *b* = 4000 s/mm^2^ along left-right diffusion-encoding direction (column ii), diffusion-weighted images (DWI) acquired at *b* = 10000 s/mm^2^ along left-right diffusion-encoding direction (column iii), fractional anisotropy (FA) maps color encoded by the primary eigenvectors (V1) from DTI (column iv), FA maps (column v) and mean kurtosis maps (column vi). Row (a) shows axial views of each map. Row (b) shows enlarged regions of interest in the internal capsule (red box) and basal ganglia (green box). Row (c) shows coronal or sagittal views for each map. Row (d) shows enlarged regions of interest in subcortical white matter (blue box) and hippocampus (yellow box). The images highlight the fine-scale gray and white matter anatomy captured by the data, both in deep and superficial brain areas.

Fig. 9 shows various maps obtained from the multi-shell dMRI scan of a *post mortem* human brain, acquired at 0.73 mm isotropic resolution with the 48ch *ex vivo* brain coil. The six columns show: *(i)* a *b* = 0 image, *(ii)* a diffusion-weighted image from the lower shell (*b* = 4000 s/mm^2^), *(iii)* the fractional anisotropy (FA) map, *(iv)* the FA map color encoded by the principal eigenvector of the diffusion tensor, *(v)* a diffusion-weighted image from the higher shell (*b* = 10000 s/mm^2^, and *(vi)* the mean kurtosis map.

For each map in the figure, an axial view is shown in row (a) and a coronal or sagittal view is shown in row (c). Row (b) shows magnified regions of interest that highlight fine anatomical detail in the striatum (red box, columns i-ii and iv-v) or in the basal ganglia and thalamus (green box, columns iii and vi). Note that the exquisite mean kurtosis contrast allows a clear delineation of internal structures such as the putamen, caudate nucleus, internal and external global palidus, and thalamus. Row (d) shows a magnified coronal view of the primary motor cortex (blue box, columns i-ii and iv-v), highlighting the presence of radial fibers, and a magnified sagittal view of the hippocampus (yellow box, columns iii and vi). These maps illustrate that, by combining the high gradient strengths available on the 3 T Connectome scanner with the high sensitivity of our *ex vivo* coil, we can collect dMRI data with high spatial resolution and high SNR, and map detailed gray and white matter anatomy, both in deep brain and near the cortical surface.

**Fig. 10.**
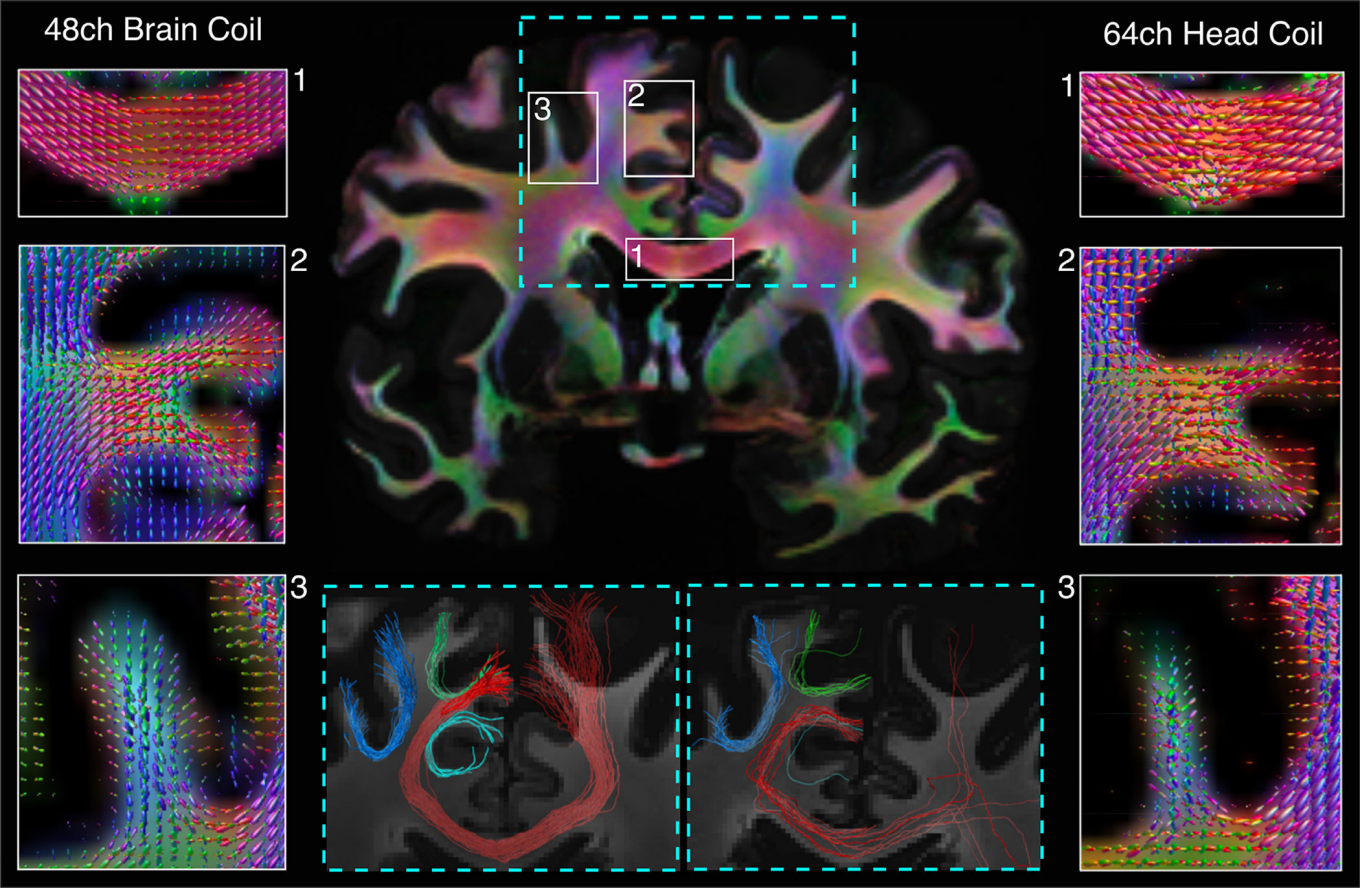
Comparison of FODs and probabilistic tractography in the same *ex vivo* human brain, between data acquired with the 48ch *ex vivo* brain coil and the 64ch head coil. A whole-brain, coronal color map of the principal diffusion directions as obtained by CSD illustrates the location of the selected regions of interest. For the three smaller boxes in the corpus callosum (1), superior frontal gyrus (2), and middle frontal gyrus (3), we compare FODs obtained with the 48ch *ex vivo* brain coil (left) and the 64ch head coil (right). For the larger box centered over the corpus callosum (outlined in blue dashed lines), we show tractography results obtained with the two coils (see blue dashed insets at the bottom of the figure). We show tractography streamlines from the corpus callosum (in red) and adjacent U-fibers (blue, light blue, green). The higher SNR achieved by the 48ch *ex vivo* brain coil resulted in less noisy FODs, which in turn improved the quality of tractography in these bundles.

Fig. 10 compares the FODs and probabilistic tractography obtained from the multi-shell dMRI data collected with the 48ch *ex vivo* brain array and the 64ch *in vivo* head array. The higher SNR achieved by the newly developed *ex vivo* coil, in both superficial and deep brain areas, yielded less noisy FODs that better captured the course of the underlying fiber bundles. This resulted in higher-quality tractography. For example, the tractogram from the 48ch brain array data included fuller bundles of trans-callosal streamlines, as well as subcortical U-shaped streamlines. The lower SNR of the 64ch head coil led to more spurious peaks in the FODs, which had a negative impact on the ability of tactography to reconstruct these bundles.

## 4. Discussion

We designed, constructed, and evaluated a 48ch *ex vivo* brain array receive coil for high-resolution and high *b*-value dMRI of a whole *ex vivo* human brain on the 3 T Connectome scanner (McNab et al., 2013; Setsompop et al., 2013). The coil was characterized by both bench tests and image metrics. Bench tests included element measurements of the coil quality factor *Q*, active detuning, geometrical decoupling, and preamplifier decoupling. MRI evaluations included measurements of the noise correlation, pixel-wise SNR, and G-factor, as well as time course stability using a brain shaped agar phantom. We demonstrated the coil’s performance in achieving high SNR with the acquisition of multi-shell 0.73 mm isotropic resolution diffusion-weighted MR images of a whole *ex vivo* brain.

### 4.1. Array coil characterization

In many applications, large channel count arrays with relatively small loop sizes such as the 54 mm loops used here are necessary to increase both reception sensitivity and encoding power. However, very small loop elements quickly lose their sample noise dominance. Under these circumstances, small elements do not translate to higher SNR acquisitions anymore. For *in vivo* imaging at 3T, this critical size is reached at about 60 mm diameter (Keil et al., 2011). In *ex vivo* brain imaging, however, loop sizes can be made substantially smaller than for *in vivo* imaging. This is attributed to the brain fixation medium, which has a higher conductivity compared to *in vivo* tissue and thus provides a higher fraction of sample noise. While the noise increases in the *ex vivo* sample, the electronic noise can be decreased by omitting *in vivo* human safety features in the coil element circuity, such as passive detuning and RF-fuses. This condition results in an enhanced *Q*_*U*_/*Q*_*L*_-ratio when using small receiver elements. Therefore, the implemented loop size of ≈54 mm provides a relatively high *Q*_*U*_/*Q*_*L*_-ratio of 5.1, outperforming most coils optimized for *in vivo* applications with loop diameters ranging from 50 mm to 65 mm from our previous studies (Janssens et al., 2012; Keil et al., 2011; 2013). As a consequence, the minimum loop diameter at which sample noise dominance is maintained decreases for imaging fixed tissue brain samples in PLP solution, allowing us to contemplate very high-density arrays for *ex vivo* sample examinations.

Despite RF electrical optimizations, the mechanical coil former is an important and critical design aspect for *ex vivo* imaging. To improve SNR, the loops were populated very close to the sample, maximizing signal reception. Thus, the completely brain-enclosing coil former with uniformly distributed loop elements guarantees nearly omni-directional signal reception from the sample (decreased sensitivity was observed with some loops aligned to be almost parallel to the magnetic field *B*_0_). However, an entirely surrounding coil array requires a split housing mechanism, which disturbs the loop layout and makes it difficult to maintain geometric decoupling at the split housing edge. Therefore, an overlapping edge structure was implemented, enabling adjacent loop elements to be geometrically decoupled across the two housing segments, while the overall array coil structure remains self-contained.

In array coil design, the central ultimate SNR is already approached with only 12 surrounding coil elements at 3 T (Wiesinger et al., 2005). Implementing higher loop element counts only yields SNR improvements at the periphery for a given geometry. Nevertheless, relative central SNR gains are achievable with tightly fitting array coils. Due to the lack of dedicated *ex vivo* receiver arrays, *in vivo* head coils are commonly used in many *ex vivo* brain studies (Iglesias et al., 2018; McNab et al., 2009; Shatil et al., 2016; 2018). However, these coils are not well suited in terms of sample fitting and SNR performance. Optimizing both the mechanical features for close fitting of samples and the RF circuitry can thus result in significant SNR gains in the brain. This implementation provides a 30% SNR increase of the 48ch coil at the phantom center when compared to the larger 64ch head coil. In addition, in the peripheral regions of the brain phantom, the tight-fitting form factor also provides favorable SNR gains, as evidenced by an almost 3-fold SNR improvement over the 64ch coil. The high SNR can be exploited to reduce the voxel size, enabling high spatial resolution MR imaging of a whole *ex vivo* brain.

The average noise correlation of 9% indicates a well decoupled array and highly independent operating receiver loops. Adjacent loops show much higher coupling values up to 36%, which can be attributed to insufficient overlap, resulting in a remaining mutual inductance and shared resistance especially in the sample voxels beneath the overlapping loop regions.

The constructed 48ch *ex vivo* brain coil shows remarkably better encoding performance when compared to the 64ch head coil. The encoding power of the 48ch coil enables approximately one additional acceleration unit, for both one-dimensional and two-dimensional accelerations, with the same noise amplification as the 64ch head coil. Improvements in G-factors are usually achieved by implementing higher channel counts on a given geometry. However, when comparing array coil formers of different sizes, similar improvements in G-factors can be achieved by (1) reducing the diameter of the coil elements at constant or even lower channel counts, and (2) positioning the coil elements in close proximity to the sample. The tight-fitting, smaller loop elements of the constructed 48ch coil provide an overall stronger spatial modulation in the signal sensitivity’s magnitude and phase. Consequently, this coil arrangement allows favorable encoding capabilities for unaliasing folded images (SENSE method) or synthesizing spatial harmonics (GRAPPA or SMASH methods). Additionally, the entirely enclosed *ex vivo* coil former of the 48ch coil leads to better spatial coverage for the aliased pixels when compared to a head array coil, which obviously has limited coverage along the inferior aspect and in the area covering the face.

When the total acceleration factor *R* is the product of the acceleration factors from two individual orthogonal phase-encoding directions, the images show less amplified noise in comparison to *R*-fold 1D accelerations. This can be attributed to the embedded sensitivity variations of the array coil being efficiently exploited in two spatial dimensions, allowing overall more favorable encoding capabilities in the image reconstruction.

Reducing scan time using parallel imaging techniques is not strictly essential when constraints on acquisition time are lifted for *ex vivo* examinations. On the other hand, 2D acquisitions are still often used despite their SNR inefficiency per unit time (Eichner et al., 2020; Miller et al., 2011). For example, mapping tissue microstructural features throughout the whole human brain involves measurements at multiple *b*-values (Huang et al., 2020), and protocol optimization may be facilitated by 2D scans acquired at resolutions on the order of 0.8 to 1 mm isotropic. For such 2D acquisitions, slice acceleration enables the excitation and measurement of multiple slices (Feinberg et al., 2010; Setsompop et al., 2018; 2012). Unlike conventional parallel imaging, which requires under-sampled data acquisition, these techniques provide acceleration by exciting the spins in multiple slices at the same time using multi-band radiofrequency pulses. These newer multi-band MR acquisitions have the SNR advantages of 3D sampling based on Fourier averaging (Larkman et al., 2001; Setsompop et al., 2012). Therefore, SNR efficiency can be improved by up to a factor of √*MB*, if the imaging parameters remain the same. In practice, however, the SNR gain is reduced locally by the SMS G-factor of the coil and globally due to changes in the sequence parameters. At higher SMS acceleration factors, shorter repetition times are employed, which deceases the level of steady-state longitudinal magnetization. Therefore, the optimal SMS acceleration factor for an MRI study is a trade-off between the benefits of the simultaneously acquired volume and the negative impact from local noise amplifications and the chosen repetition times.

The SNR recovery achieved by the SMS method is highly advantageous for dMRI, which normally suffers from low signal strength. Therefore, it is advantageous for *ex vivo* array coils to provide a high encoding capability for SMS in order to accommodate modern acquisition techniques. Commonly used *in vivo* head coils do not optimally fulfill this requirement for SMS *ex vivo* scans, as they lack enough elements in the *z*-direction. The radially surrounding, *z*-directional, stacked elements of the constructed coil provide favorable spatial coverage for SMS image encoding, allowing the separation of multiple collapsed slices. In the case of an *MB* = 8 acceleration scheme, the combination of the enhanced SMS encoding power and the increased baseline SNR of the 48ch coil, would provide an up to 4.5-fold SNR improvement when compared to the 64ch head coil.

### 4.2. *Diffusion imaging in* ex vivo *brain*

Previous work comparing *ex vivo* dMRI to optical imaging suggests that high spatial resolution (1 mm or higher) improves the accuracy of dMRI-derived axonal orientation estimates, and may have a greater impact than high angular resolution or ultra-high *b*-values (Jones et al., 2020). While dMRI acquisitions with sub-mm resolution have been feasible in pre-clinical scanners, for smaller samples, they have remained a challenge for whole human brains. The 48ch *ex vivo* brain coil that we have developed here has enabled us to collect high-resolution (0.73 mm isotropic) dMRI data from a whole *ex vivo* human brain. We have shown that this coil achieves higher SNR than a 64ch *in vivo* head coil throughout the brain, leading to improved delineation of brain circuitry with dMRI tractography. The 48ch brain coil exhibits the highest SNR gains in the peripheral area of the imaged sample, which will be of particular utility for imaging fiber architecture in the cortex with high precision. Examples of this in our results are the radial fibers in the primary motor cortex in Fig. 9 (column iv, row d), as well as the subcortical U-shaped tracts in Fig. 10. Future work in laminar microstructure in the cortex will greatly benefit from this coil. The high sensitivity of the coil enables high spatial resolution in combination with very high b-values, as we have shown with our preliminary results at *b* = 10000 s/mm^2.^ Indeed, mean kurtosis maps obtained from the acquired data reveal exquisite contrast that captures the internal structure of the basal ganglia and hippocampus.

The coil presented here paves the way for sub-mm resolution *ex vivo* dMRI on whole human brains at the high *b*-values accessible on the 3 T Connectome scanner. This capability will allow us to map the connectional anatomy and microstructure of the human brain at unprecedented resolutions, as well as provide reference data for evaluating *in vivo* dMRI scans to gain deeper insight into human brain structure at multiple scales. Currently, 3D EPI suffers from ghosting artifacts when both spatial resolution and gradient amplitude are increased up to the limit of the Connectome scanner’s capacity. In future work, we will address this issue by combining appropriate *k*-space reconstruction techniques (Ramos-Llorden et al., 2021) and the use of an additional field monitoring camera system (Mahmutovic et al., 2021). We expect this novel coil design, in combination with the current 3 T Connectome scanner equipped with 300 mT/m gradient strengths and next-generation gradient system planned for the Connectome 2.0 project (Yendiki et al., 2020), to advance our understanding of human brain circuitry in health and disease.

## 5. Conclusion

A 48ch close-fitting receive array coil for dMRI of whole *ex vivo* human brains at 3 T was designed, constructed, and tested with a brain-shaped phantom and an *ex vivo* brain. We characterized the coil with unloaded-to-loaded *Q*-ratio, noise correlation, SNR, G-factor, SMS G-factor and stability measurements in comparison to a 64ch whole-head *in vivo* coil. Compared to *in vivo* array coils, smaller loop sizes can be used for *ex vivo* brain samples due to increased loading characteristics of the fixed brain tissue. This allows the design of high-channel count arrays, improving both peripheral SNR and encoding performance for accelerated imaging. Due to the high SNR and parallelism, the designed coil is well-suited for high-resolution, high *b*-value *ex vivo* dMRI acquisitions and will enable to map the connectomics and microstructure of the human brain at multiple scales.

## Footnotes

1 under development and not commercially available in the U.S. and its future availability cannot be assured.

## Abbreviations:

48ch: 48-channel
64ch: 64-channel
ABS: acrylonitrile butadiene styrene
BW: bandwidth
CAD: computer aided design
CSD: constrained spherical deconvolution
dMRI: Diffusion MRI
DKI: diffusion kurtosis imaging
DTI: diffusion tensor imaging
DWI: Diffusion-Weighted Imaging
DWMRI: Diffusion-weighted Magnetic Resonance Imaging
EPI: echo planar imaging
EPROM: Erasable Programmable Read-Only Memory
F: flip angle
FA: fractional anisotropy
FOD: fiber orientation distribution
FOV: field of view
M: matrix
MRI: Magnetic Resonance Imaging
MSMT-CSD: multi-shell, multi-tissue, constrained, spherical deconvolution
PC: polycarbonate
PCB: printed circuit board
PD: proton density
PLP: periodate-lysine-paraformaldehyde
RF: radio frequency
ROI: region of interest
SMS: simultaneous multislice
SNR: signal-to-noise-ratio
TE: echo time
TR: repetition time
VNA: vector network analyzer
